# Effects of non-fasting molting on performance, oxidative stress, intestinal morphology, and liver health of laying hens

**DOI:** 10.3389/fvets.2023.1100152

**Published:** 2023-02-28

**Authors:** Meng Lei, Lei Shi, Chenxuan Huang, Yawei Yang, Bo Zhang, Jianshe Zhang, Yifan Chen, Dehe Wang, Erying Hao, Fengling Xuan, Hui Chen

**Affiliations:** ^1^College of Animal Science and Technology, Hebei Agricultural University, Baoding, China; ^2^Yangling Hongyan Molting Research Institute, Yangling, China; ^3^Agricultural and Animal Husbandry Technology Extension Station in Tong Town, Yulin, Shaanxi, China; ^4^Gaocheng District Animal Husbandry Work Station, Shijiazhuang, China

**Keywords:** non-fasting method, fasting method, production performance, oxidative stress, intestinal morphology, serum biochemical indicators, liver tissue morphology

## Abstract

Animal welfare concerns in laying-hen production facilities have necessitated research on alternative strategies for improving egg production and hen health. At present, most laying-hen facilities in China use the fasting method, but with international emphasis on animal welfare, scholars have begun to find ways to improve production efficiency while ensuring animal welfare standards are adhered to. Therefore, this study investigated the effects of non-fasting molting on production performance, oxidative stress, intestinal morphology, and liver health of laying hens. A total of 180 healthy 90-week-old Dawu Jinfeng laying hens with similar body weights and laying rates (76 ± 2%) were randomly divided into three groups, with five replicates per group and 12 hens per replicate. The hens in the experimental group (NF) were molted using the non-fasting method, the negative control group (C) was not treated with centralized molting, and the positive control group (F) was molted using the fasting method. The results showed that: (1) During the molting period, the laying rate in the NF group (10.58%) decreased and was significantly lower than that in the other two groups (*P* < 0.05). During the secondary laying peak period, the laying rate in the NF group was highest (89.71%); significantly higher than that in the C group (*P* < 0.05). (2) During the molting period, compared to the C group, the NF group showed a significant decrease and increase in the total antioxidant capacity (T-AOC) and superoxide dismutase (T-SOD) activity, respectively (*P* < 0.05). During the secondary laying peak period, the T-SOD activity of the NF group was significantly lower than that of the C group (*P* < 0.05). (3) During the molting period, the villus height (VH) and the ratios of VH to crypt depth (V/C) of the duodenum, jejunum, and ileum in the NF group were significantly lower than those in the C group (*P* < 0.05). At the secondary laying peak period, the jejunum V/C was significantly higher than that in the C group (*P* < 0.05), whereas in the duodenum and ileum it increased but not significantly (*P* > 0.05). (4) During the molting period, serum glutathione transaminase (AST) and glutathione alanine transaminase (ALT) activities were significantly higher (*P* < 0.05), and very low-density lipoprotein (VLDL) content and liver weight were significantly lower (*P* < 0.05) in the non-fasted and fasted groups. However, there was a low degree of liver injury (cell boundary still visible) in the NF group. At the secondary laying peak period, there was no significant difference (*P* > 0.05) in the indices among the three groups and the liver returned to normal. In summary, non-fasting molting can improve the production performance of laying hens in the later stages, ensure the welfare and health of animals, and provide a theoretical basis for the efficient production of laying hens.

## 1. Introduction

Poultry molting is a natural physiological phenomenon. However, owing to the inconsistency of molting time and speed, the peak period in secondary egg production, which affects the production efficiency of laying hens, is challenging to identify. In order to shorten the molting time, extend the service life, and therefore maximize the benefits of laying hens to industry, artificial forced-molting techniques have received widespread attention. Artificial forced-molting techniques are used by humans to induce tissue metabolism disorder, prompting the synchronous weight loss and cessation of egg laying, and then restoring the process of egg laying (1). At present, most laying-hen facilities in China use the fasting method, but with international emphasis on animal welfare, scholars have begun to find ways to improve production efficiency while ensuring animal welfare standards are adhered to. Zhang et al. (2) showed that artificial forced-molting techniques could delay the aging of laying hens and redevelop their brain, reproductive system, and other tissues and organs. Studies have shown that the production performance of laying hens, such as the egg production rate and feed conversion rate, improves after molting (3, 4). Bozkurt et al. (5) showed that eggshell thickness, eggshell strength, and the Haugh unit significantly improved after molting. Furthermore, a large number of studies have shown that artificial forced-molting techniques can prolong the laying period of hens and effectively improve laying performance and egg quality (6–9). However, there are few studies on the functional changes in various tissues of laying hens during molting. Therefore, in this experiment, the non-fasting method was used to molt laying hens and these were compared with a negative control group (no centralized molting treatment) and a positive control group (fasting molting treatment) to observe and analyze its effects on production performance, oxidative stress, intestinal morphology, and liver health, in order to provide a theoretical basis for the application of the non-fasting molting technique.

## 2. Materials and methods

### 2.1. Experimental animals

A total of 180 90-week-old Dawu Jinfeng laying hens with a good mental state, similar body weights, and a similar laying rate (76 ± 2%) were randomly divided into three groups with five replicates per group and 12 laying hens per replicate. The experiment was conducted in a two-level cage in an environmentally controlled chamber at Hebei Agricultural University. The experimental group (NF) was treated with the non-fasting method, the negative control group (C) was not treated with centralized molting, and the positive control group (F) was treated with the fasting method. The experiment lasted for 90 days: 7 pre-experimental and 83 experimental days.

### 2.2. Test scheme

The laying hens in the NF group were fed a replacement diet (50 g/hen per day) throughout the molting period and the water supply was normal. The weight loss rate of laying hens was observed and recorded. When the weight loss rate reached ~25%, the basal diet was resumed (gradually increasing the feed amount to 120 g/hen per day and then changing to free feeding). The chamber was maintained under constant light conditions of 8 h/d (8:00–16:00) during the molting period, and the light was gradually increased to 16 h/d (8:00–22:00) after the basal diet was restored.

The laying hens in the F group were fasted throughout the molting period, water was stopped on day 8 and 9, and free drinking water was allowed at other times. When the body weight decreased to ~25%, the basal diet was resumed (gradually increasing the feeding amount to 120 g/hen per day and then changing to free feeding). During the molting period, the henhouse was maintained under light conditions of 8 h/d (08:00–16:00), which was gradually increased to 16 h/d (08:00–22:00) after the basal diet was reintroduced.

The C group was fed a basal diet without any treatment, with normal drinking water and light maintenance for 16 h/d (8:00–22:00). The composition and nutritional levels of the basal and feather replacement diet are shown in Table 1.

**Table 1:** Ingredients and chemical composition of basal diet.

**Items**	**Content**	
	**Basal diet**	**Diet of molting period**
**Ingredients**		
Corn	62.50	54.00
Soybean meal	25.20	
Corn gluten meal	0.50	
Rice bran		16.00
Wheat bran		24.00
Limestone	9.30	4.10
CaHPO_4_	0.90	0.70
DL-Met	0.10	
Premix^a^	1.00	1.00
NaCl	0.50	0.20
Total	100.00	100.00
**Nutrient levels** ^b^		
ME/(MJ/kg)	10.91	10.75
CP	15.98	10.51
CF	2.10	3.78
Ca	3.63	1.77
P	0.27	0.87
Lys	0.80	0.36
Met	0.45	0.17

^a^The premix provided the following per kg of the diet: VA 11 000 IU, VD33 000 IU, VE 12 IU, VK 3 mg, VB12 mg, VB26 mg, VB63 mg, VB120.012 mg, biotin 0.04 mg, folic acid 0.6 mg, niacin 20 mg, pantothenic acid 10 mg, ethoxyquin 0.4 mg, Cu 10 mg, I 0.35 mg, Fe 65 mg, Mn 120 mg, Se 0.25 mg, and Zn 65 mg.

^b^Nutrient levels were all calculated values.

### 2.3. Sample collection

In order to observe the physiological changes of laying hens during the whole molting process, the whole experiment period was divided into three stages: stage I, the laying rate during molting was 0% and the weight loss rate was ~25%; stage II, the laying rate was ~50% after reintroducing the basal diet; and stage III, the egg production rate was stable at more than 85% after reintroducing the basal diet; that is, the secondary laying peak was reached.

Two laying hens were randomly selected from each replicate at each of the three stages (I–III). Blood was collected from the wing vein, centrifuged at 3,000 r/min, and the supernatant was collected and stored at −20°C. At the same time, one laying hens was randomly selected for slaughter test.

#### 2.3.1. Production performance

Under the same management, the number of eggs laid during the molting period (from the beginning of the experiment to the time when the egg production rate dropped to 0% and the weight loss rate was ~25%) and the secondary laying peak period (2 weeks after the egg production rate reached 85%) was recorded in units of repetition. The egg production rate was calculated, and the days required for the egg production rate to drop to 0% were observed.

During the molting period, the body weights and number of days in the molting period (laying rate = 0%, weight loss rate ~25%) of the laying hens in the NF and F groups were recorded.

#### 2.3.2. Serum antioxidant indices

The levels of corticosterone (CROT), total antioxidant capacity (T-AOC), malondialdehyde (MDA), and superoxide dismutase (T-SOD) in the serum were measured using kits. The CROT, T-AOC, and MDA kits were purchased from Shanghai Jianglai Biotechnology Co. Ltd. The T-SOD kit was purchased from Nanjing Jiancheng Bioengineering Institute.

#### 2.3.3. Intestinal morphology

One laying hen was randomly selected from each replicate at each of the three stages (I–III). The intestinal segments of ~3 cm were cut from the middle of the duodenum, jejunum, and ileum, and stored in 4% paraformaldehyde solution to prepare intestinal tissue sections and hematoxylin and eosin (HE) staining. Villus height (VH) and crypt depth (CD) of the intestinal mucosa were measured, and the ratio of VH to CD (V/C) was calculated.

#### 2.3.4. Serum biochemical indices

The levels of aspartate aminotransferase (AST), alanine aminotransferase (ALT), and very low-density lipoprotein (VLDL) in the serum were measured using specific kits purchased from Nanjing Jiancheng Bioengineering Institute.

#### 2.3.5. Liver tissue morphology

One laying hen was randomly selected from each replicate at each of the three stages (I–III) and was slaughtered after bloodletting. The liver was separated non-destructively, the gallbladder was removed, weighed, and ~1 cm^2^ of the left liver was cut and immersed in a pre-prepared 4% paraformaldehyde solution. Liver tissue sections were prepared and HE stained to observe the degree of liver damage.

#### 2.3.6. Serum immune index

Serum levels of immunoglobulin A (IgA), immunoglobulin M (IgM), and immunoglobulin G (IgG) were measured using an ELISA kit purchased from Shanghai Jianglai Biotechnology Co., Ltd.

### 2.4. Statistical analysis

The experimental data were initially sorted using Excel 2010, and then one-way analysis of variance (ANOVA) was performed using SPSS software (version 19.0). Duncan's method was used for multiple comparisons between the groups. *P* < 0.05 indicated that the difference was significant.

## 3. Results

### 3.1. Effects of non-fasting molting on production performance

As shown in Table 2, the egg production rate of the NF group was the lowest during the molting period, which was significantly lower than that of the F and C groups (*P* < 0.05). During the secondary laying peak period, the egg production rate of the NF and F groups increased, and was significantly higher than that of the C group (*P* < 0.05). The NF group had the longest molting period of 40 days, whereas the F group molted faster and molting was completed within 10 days.

**Table 2:** Effects of non-fasting molting on the performance of laying hens.

**Indices**	**Groups**			***P*-value**
	**C**	**F**	**NF**	
Egg production rate during molting period	75.00 ± 1.64^a^	14.74 ± 0.91^b^	10.58 ± 1.17^c^	< 0.001
Egg production rate during secondary laying peak period	70.85 ± 1.98^b^	89.31 ± 1.45^a^	89.71 ± 1.64^a^	< 0.001
Molting period days	-	10	40	-

C, not treated with centralized molting; F, fasting molting; NF, non-fasting molting.

^a−*c*^P < 0.05. Means within a column followed by different superscript letters differ significantly.

### 3.2. Effect of non-fasting molting on oxidative stress

As shown in Table 3, in stage I, compared with the C group, the T-SOD activity of the NF and F groups was significantly increased (*P* < 0.05), and the T-AOC was significantly decreased (*P* < 0.05). In stage II, with the reintroduction of the basal diet, the T-SOD activity of the NF and F groups decreased but was still higher than that of the C group (*P* < 0.05). The T-AOC index was significantly higher in the NF group than in the other two groups (*P* < 0.05). In stage III, the T-SOD activity in the NF and F groups was significantly lower than that in the C group (*P* < 0.05), however there was no significant difference in the other oxidative stress indicators among the three groups (*P* > 0.05).

**Table 3:** Effects of non-fasting molting on oxidative stress in laying hens.

**Testing stages**	**Indices**	**Groups**			***P*-value**
		**C**	**F**	**NF**	
Stage I	CORT (ng/mL)	75.38 ± 18.62	87.21 ± 11.13	85.71 ± 17.32	0.469
	T-AOC (U/mL)	19.36 ± 1.28^a^	16.94 ± 1.81^b^	16.53 ± 1.59^b^	0.031
	MDA (nmol/mL)	8.22 ± 1.12	9.76 ± 2.25	9.12 ± 1.77	0.412
	T-SOD (U/mL)	213.81 ± 21.74^b^	332.06 ± 15.61^a^	308.26 ± 37.35^a^	< 0.001
Stage II	CORT (ng/mL)	79.55 ± 5.03	82.03 ± 18.67	79.40 ± 16.65	0.955
	T-AOC (U/mL)	18.51 ± 0.76^b^	18.39 ± 2.19^b^	22.59 ± 3.22^a^	0.021
	MDA (nmol/mL)	8.95 ± 0.67	9.04 ± 0.78	8.09 ± 1.08	0.166
	T-SOD (U/mL)	236.44 ± 15.75^b^	309.46 ± 6.03^a^	295.86 ± 31.82^a^	0.010
Stage III	CORT (ng/mL)	77.33 ± 14.90	80.52 ± 19.19	77.39 ± 21.65	0.951
	T-AOC (U/mL)	18.30 ± 2.09	19.64 ± 1.77	19.82 ± 1.57	0.295
	MDA (nmol/mL)	8.48 ± 1.30	7.97 ± 0.75	8.04 ± 1.74	0.804
	T-SOD (U/mL)	218.94 ± 38.33^a^	176.88 ± 2.27^b^	162.39 ± 24.59^b^	0.012

C, not treated with centralized molting; F, fasting molting; NF, non-fasting molting.

^a, b^P < 0.05. Means within a column followed by different superscript letters differ significantly.

CORT, corticosterone; T-AOC, total antioxidant capacity; MDA, malondialdehyde; SOD, superoxide dismutase.

### 3.3. Effect of non-fasting molting on intestinal morphology

As shown in Table 4, in stage I, the VH and V/C of the duodenum, jejunum, and ileum in the NF and F groups were significantly decreased in both groups (*P* < 0.05). In stage II, the VH and V/C of the duodenum in the NF and F groups were significantly lower than those in the C group (*P* < 0.05). The VH and V/C of the jejunum in the NF group were significantly lower than those in the F group (*P* < 0.05). The V/C of the ileum in the NF group was significantly higher than that in the F and C groups (*P* < 0.05). In stage III, the VH of the duodenum in the NF and F groups was significantly lower than that in the C group (*P* < 0.05), and the V/C ratio in the F group was significantly lower than that in the other two groups (*P* < 0.05). The VH and V/C of the jejunum in the NF and F groups were significantly higher than those in the C group (*P* < 0.05). The V/C of the ileum in the NF group was significantly lower than that in the F group (*P* < 0.05).

**Table 4:** Effects of non-fasting molting on intestinal morphology of laying hens.

**Testing stages**	**Indices**	**Groups**			***P*-value**
		**C**	**F**	**NF**	
Stage I	Duodenum				
	VH/μm	1,610.40 ± 286.12^a^	1,260.98 ± 267.97^b^	1,074.87 ± 125.06^c^	< 0.001
	CD/μm	237.82 ± 37.88^c^	310.96 ± 34.66^a^	287.51 ± 30.58^b^	< 0.001
	V/C	6.90 ± 1.59^a^	4.08 ± 0.86^b^	3.77 ± 0.54^b^	< 0.001
	Jejunum				
	VH/μm	930.34 ± 206.32^a^	648.57 ± 166.03^b^	697.10 ± 176.09^b^	< 0.001
	CD/μm	176.33 ± 44.30^b^	224.55 ± 40.67^a^	186.75 ± 32.06^b^	< 0.001
	V/C	5.47 ± 1.35^a^	2.93 ± 0.73^c^	3.78 ± 0.86^b^	< 0.001
	Ileum				
	VH/μm	857.07 ± 154.14^a^	543.87 ± 108.96^b^	431.58 ± 122.30^c^	< 0.001
	CD/μm	157.96 ± 24.77^b^	197.36 ± 21.59^a^	168.50 ± 32.74^b^	< 0.001
	V/C	5.53 ± 1.34^a^	2.77 ± 0.55^b^	2.62 ± 0.77^b^	< 0.001
Stage II	Duodenum				
	VH/μm	1,596.67 ± 258.30^a^	1,401.04 ± 378.62^b^	1,044.07 ± 181.30^c^	< 0.001
	CD/μm	222.46 ± 25.59^b^	317.38 ± 59.19^a^	221.63 ± 29.01^b^	< 0.001
	V/C	7.27 ± 1.46^a^	4.47 ± 1.19^b^	4.78 ± 0.99^b^	< 0.001
	Jejunum				
	VH/μm	918.60 ± 255.50^b^	1,340.56 ± 253.71^a^	790.85 ± 198.12^b^	< 0.001
	CD/μm	167.85 ± 28.21	157.32 ± 41.29	156.07 ± 64.37	0.605
	V/C	5.54 ± 1.54^b^	9.09 ± 2.74^a^	5.45 ± 1.35^b^	< 0.001
	Ileum				
	VH/μm	852.98 ± 132.91	871.09 ± 171.47	772.04 ± 241.58	0.124
	CD/μm	145.77 ± 24.88^a^	150.46 ± 28.74^a^	94.25 ± 13.20^b^	< 0.001
	V/C	5.97 ± 1.11^b^	5.88 ± 1.05^b^	8.15 ± 2.12^a^	< 0.001
Stage III	Duodenum				
	VH/μm	1,574.49 ± 290.33^a^	1,334.80 ± 194.79^b^	1,389.72 ± 191.92^b^	0.001
	CD/μm	248.18 ± 29.60^a^	264.20 ± 28.24^a^	219.93 ± 39.63^b^	< 0.001
	V/C	6.34 ± 0.89^a^	5.11 ± 0.96^b^	6.40 ± 0.77^a^	< 0.001
	Jejunum				
	VH/μm	964.51 ± 125.22^b^	1,084.58 ± 111.90^a^	1,123.99 ± 250.26^a^	0.004
	CD/μm	193.28 ± 48.03^a^	129.87 ± 21.46^b^	127.43 ± 38.00^b^	< 0.001
	V/C	5.24 ± 1.26^b^	8.60 ± 1.83^a^	9.86 ± 4.04^a^	< 0.001
	Ileum				
	VH/μm	897.38 ± 193.99^a^	835.04 ± 70.90^a^	731.52 ± 87.70^b^	< 0.001
	CD/μm	156.42 ± 38.59^a^	114.17 ± 22.36^b^	115.24 ± 20.69^b^	< 0.001
	V/C	6.04 ± 1.90^b^	7.54 ± 1.42^a^	6.57 ± 1.55^b^	0.005

C, not treated with centralized molting; F, fasting molting; NF, non-fasting molting.

^a−*c*^P < 0.05. Means within a column followed by different superscript letters differ significantly.

VH, Villus height; CD, Crypt depth; V/C, villus height/crypt depth ratio.

### 3.4. Effect of non-fasting molting on serum biochemical indices

Table 5 shows that in stage I, the VLDL content of the NF and F groups was significantly lower than that in the C group (*P* < 0.05). The AST and ALT levels were significantly higher in the F group than in the NF and C groups (*P* < 0.05). In stage II, the activity of AST in the NF group was significantly lower than that in the F and C groups (*P* < 0.05). In stage III, the liver function of laying hens gradually recovered, and there were no significant differences in AST, ALT, and VLDL levels among the three groups (*P* > 0.05).

**Table 5:** Effects of non-fasting molting on serum biochemical indexes of laying hens.

**Testing stages**	**Indices**	**Groups**			***P*-value**
		**C**	**F**	**NF**	
Stage I	AST (U/L)	27.32 ± 1.18^c^	44.34 ± 2.39^a^	41.16 ± 1.59^b^	< 0.001
	ALT (U/L)	0.74 ± 0.16^b^	1.01 ± 0.10^a^	0.86 ± 0.04^b^	0.002
	VLDL (mmol/L)	11.02 ± 1.24^a^	7.93 ± 1.21^b^	7.91 ± 1.47^b^	0.004
Stage II	AST (U/L)	27.49 ± 2.92^a^	25.99 ± 3.73^a^	21.49 ± 1.23^b^	0.026
	ALT (U/L)	0.70 ± 0.13	0.64 ± 0.11	0.75 ± 0.03	0.100
	VLDL (mmol/L)	10.21 ± 0.48	10.74 ± 1.35	11.68 ± 1.71	0.285
Stage III	AST (U/L)	27.04 ± 1.17	22.71 ± 2.72	24.42 ± 3.59	0.108
	ALT (U/L)	0.71 ± 0.03	0.63 ± 0.10	0.68 ± 0.12	0.424
	VLDL (mmol/L)	10.44 ± 2.03	12.08 ± 1.98	12.06 ± 1.09	0.143

C, not treated with centralized molting; F, fasting molting; NF, non-fasting molting.

^a−*c*^P < 0.05. Means within a column followed by different superscript letters differ significantly.

AST, Glutamic-oxalacetic transaminase; ALT, Glutamic pyruvic transaminase; VLDL, Very low density lipoprotein.

### 3.5. Effect of non-fasting molting on liver tissue morphology

As shown in Table 6, in stage I, the liver weights of hens in the NF and F groups were significantly lower than that in the C group (*P* < 0.05). After resuming the basal diet, the liver weights of the laying hens in the NF and F groups gradually increased, and there was no longer a significant difference in the liver weights of the three groups from stage II to stage III (*P* > 0.05).

**Table 6:** Effects of non-fasting molting on liver weight of laying hens.

**Testing stages**	**Groups**			***P*-value**
	**C**	**F**	**NF**	
Stage I	42.54 ± 4.57^a^	21.88 ± 3.90^b^	16.76 ± 2.52^b^	< 0.001
Stage II	39.83 ± 2.87	35.94 ± 4.45	46.45 ± 9.67	0.104
Stage III	36.82 ± 3.09	41.28 ± 5.12	36.46 ± 2.38	0.115

C, not treated with centralized molting; F, fasting molting; NF, non-fasting molting.

^a, b^P < 0.05. Means within a column followed by different superscript letters differ significantly.

**Figure 1:**
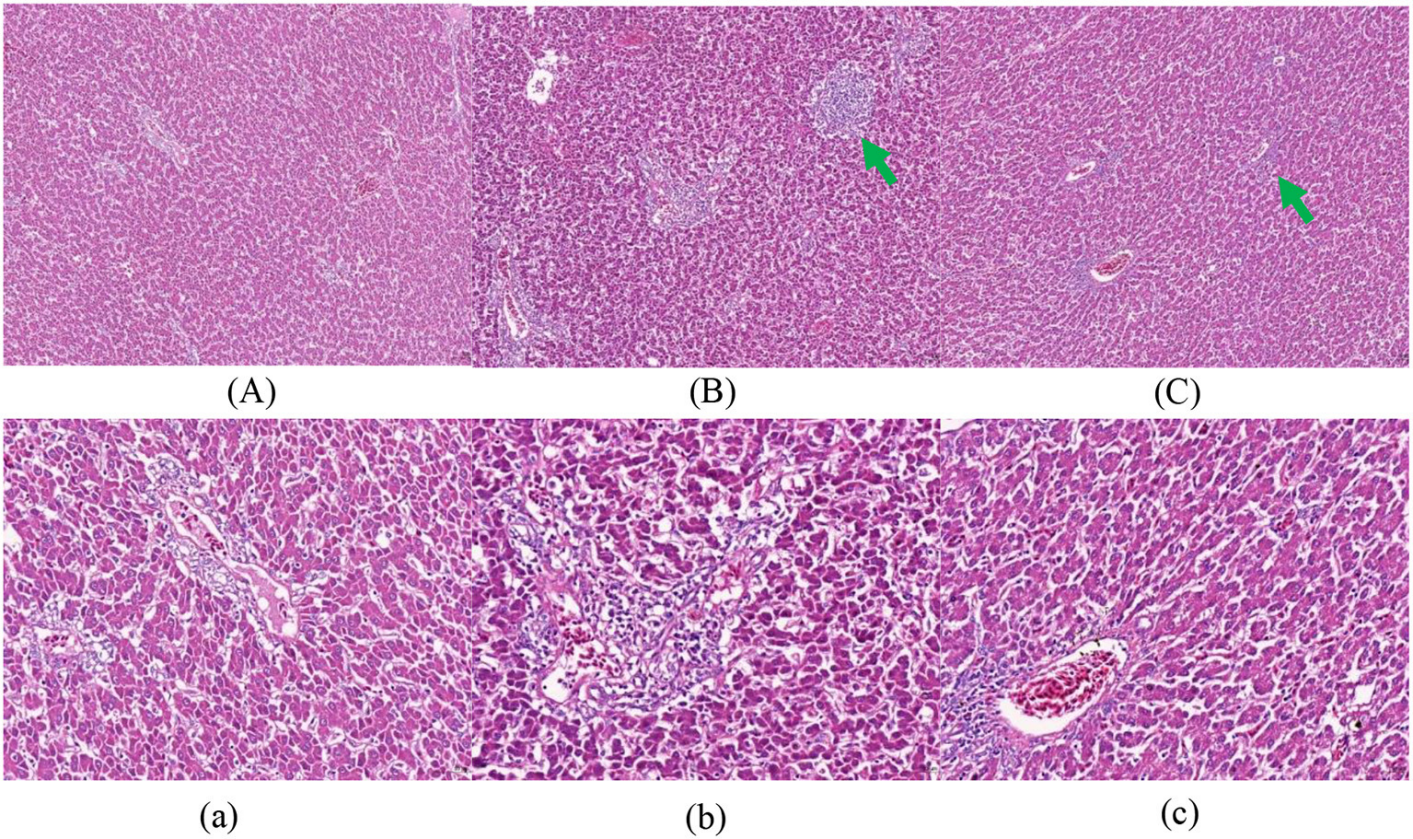
The effect of non-fasting molting on liver tissue morphology (stage I). **(A, a)** Not treated with centralized molting; **(B, b)** fasting molting; **(C, c)** non-fasting molting. Upper-case letters are ×100, lower-case letters are ×400. Green arrows represent hepatocellular edema and blue arrows indicate the occurrence of steatosis.

**Figure 2:**
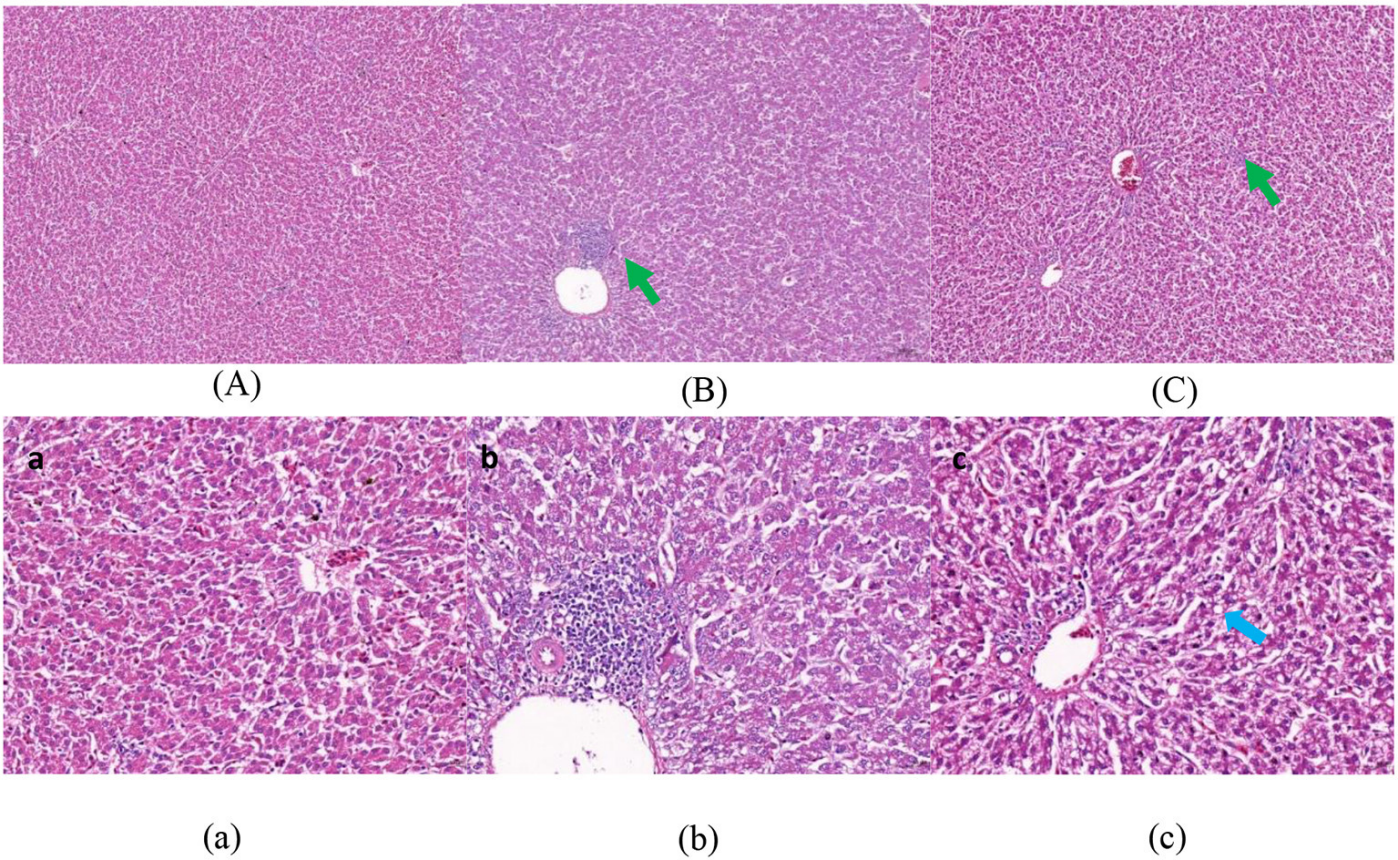
The effect of non-fasting molting on the morphology of liver tissue (stage II). **(A, a)** Not treated with centralized molting; **(B, b)** fasting molting; **(C, c)** non-fasting molting. Upper-case letters are ×100, lower-case letters are ×400. Green arrows represent hepatocellular edema and blue arrows indicate the occurrence of steatosis.

**Figure 3:**
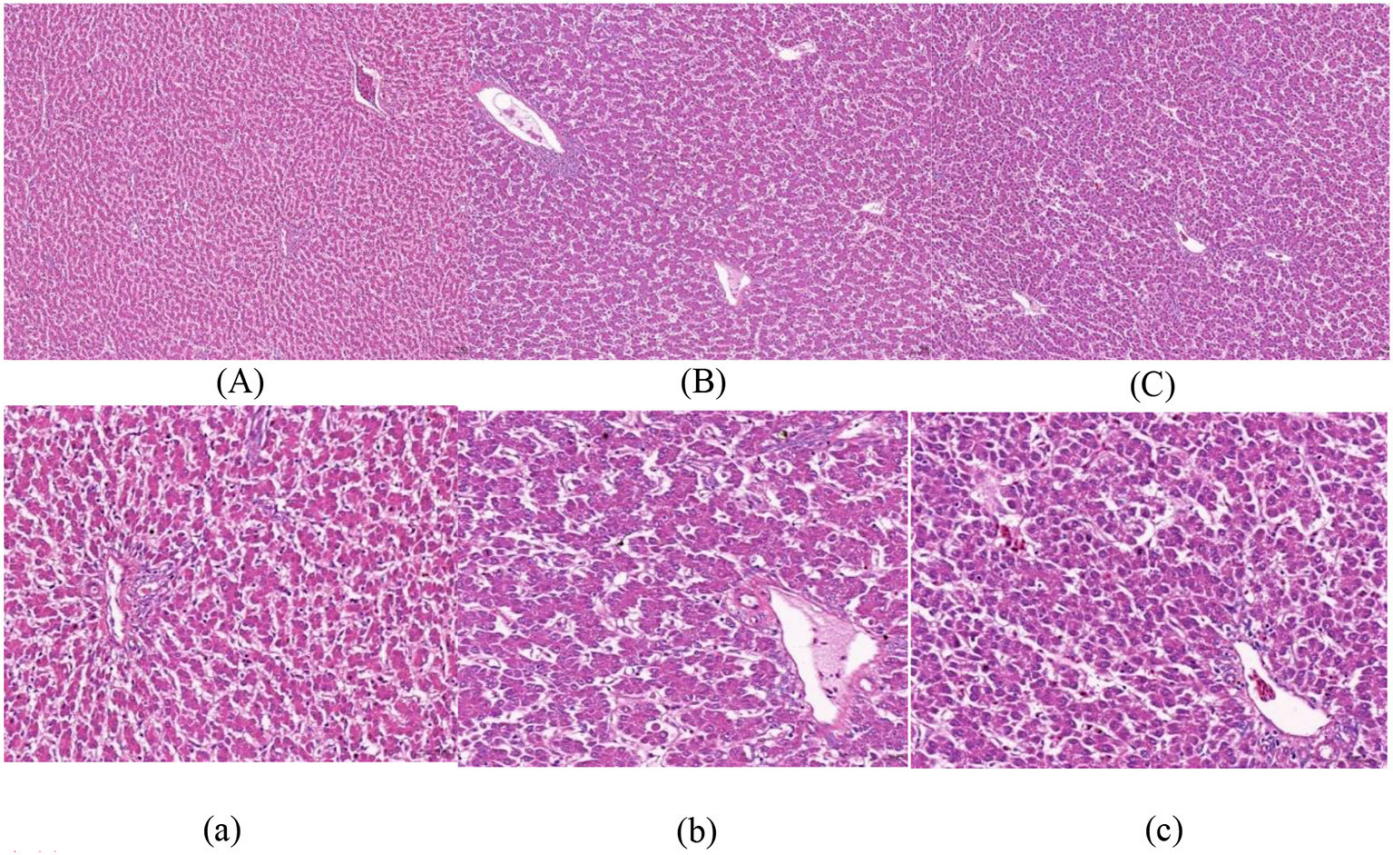
The effect of non-fasting molting on liver tissue morphology (stage III). **(A, a)** Not treated with centralized molting; **(B, b)** fasting molting; **(C, c)** non-fasting molting. Upper-case letters are ×100, lower-case letters are ×400. Green arrows represent hepatocellular edema and blue arrows indicate the occurrence of steatosis.

Figures 1–3 show that in stage I, the livers of all hens were damaged to some extent, among which the damage in the F group was more serious, with obvious swelling of hepatocytes, unclear intercellular boundaries, inflammatory cell infiltration, dilated and congested liver sinusoids, and slight cytoplasmic infection. The hepatocytes in the NF group increased in size, but there were still intercellular boundaries, and the cytoplasm began to show signs of infection. The liver gradually recovered as the level of nutrition increased. In stage II, the hepatocytes in the NF and F groups of laying hens were still slightly swollen, and in stage III, the liver in the NF and F groups had almost completely recovered.

### 3.6. Effect of non-fasting molting on immune performance

As shown in Table 7, in stage I, the IgG content of laying hens in the NF and F groups decreased significantly (*P* < 0.05), whereas in stage II, IgG gradually increased, and there were no significant differences in IgG, IgA, and IgM among NF group, F group, and C group up to stage III (*P* > 0.05).

**Table 7:** Effects of non-fasting molting on immune performance of laying hens.

**Testing stages**	**Indices**	**Groups**			***P*-value**
		**C**	**F**	**NF**	
Stage I	IgA (μg/mL)	206.47 ± 33.72	165.68 ± 21.64	173.95 ± 32.24	0.113
	IgM (μg/mL)	485.99 ± 50.74	449.26 ± 15.84	455.16 ± 14.88	0.190
	IgG (μg/mL)	1,415.25 ± 245.31^a^	989.76 ± 119.78^b^	1,005.53 ± 130.98^b^	0.003
Stage II	IgA (μg/mL)	197.40 ± 7.25	213.20 ± 3.68	226.60 ± 20.92	0.246
	IgM (μg/mL)	453.13 ± 23.14	475.59 ± 74.38	490.43 ± 38.84	0.520
	IgG (μg/mL)	1,200.66 ± 115.12	1,320.23 ± 205.76	1,361.13 ± 158.83	0.373
Stage III	IgA (μg/mL)	215.25 ± 31.24	225.18 ± 29.63	229.20 ± 26.38	0.644
	IgM (μg/mL)	467.19 ± 36.40	469.26 ± 56.92	477.96 ± 34.91	0.860
	IgG (μg/mL)	1,321.78 ± 238.07	1,477.93 ± 238.07	1,497.47 ± 246.91	0.394

C, not treated with centralized molting; F, fasting molting; NF, non-fasting molting.

^a, b^P < 0.05. Means within a column followed by different superscript letters differ significantly.

IgA, immunoglobulin A; IgM, immunoglobulin M; IgG, immunoglobulin G.

## 4. Discussion

Studies have shown that a weight loss rate of 25–35% is appropriate for hens during the molting period (10), and either too much or too little weight loss will affect later production performance. Berry and Brake (11) showed that a weight loss rate of 25–30% during the molting period can result in better egg production performance after molting. In this experiment, the 25% weight loss rate and egg production cessation were used as markers of the molting period to obtain better post-molting production performance. Tiwary et al. (7) showed that the egg production rate of hens significantly increased after molting and this was supported in a study by Onbaşilar et al. (12). Gongruttananun et al. (13) found that by comparing the effect of different molting methods on the egg production rate of late-laying hens, compared with the control group, different molting methods can improve the laying performance of hens in later periods. In this study, the results showed that the laying rate of hens in the NF group decreased significantly during the molting period and then gradually recovered as the basal diet was reintroduced. During the secondary laying peak period, the laying rate of hens in the NF group was significantly higher than that in the C group. Brake and Thaxton (14) showed that the improvement in egg production performance after molting was associated with a reduction in fat and an increase in tissue efficiency after the redevelopment of reproductive organs. Additionally, in the present study, we found that the period of non-fasting molting lasted 40 days, which was longer than the period of fasting molting, and this finding was attributed to the high energy of the diet provided during the molting period or the high feeding amount. However, this aspect will require further investigation.

Prolonged starvation disrupts the homeostasis of oxygen free radicals in organisms, leading to oxidative stress (15). MAD is an end product of oxidative reactions and has toxic effects on cells, whereas high levels of CORT are considered to be an indicator of stress in chickens. Studies have shown that the levels of CORT and MAD are significantly increased in laying hens stimulated by starvation during the molting period (9, 16). Furthermore, Andreatti Filho et al. (17) found that plasma CORT levels were increased in the group fed wheat bran during the molting period but these did not reach significant levels. The results of this experiment showed that CORT and MAD levels were elevated in the NF group during stage I but did not reach significant levels compared to the C group, presumably because of the high nutritional level of the diet during the molting period, and the low stress on the hens that had adapted to the experimental environment. At the same time, the content of CORT and MAD in the NF group was lower than that in the F group, indicating that the stress on the laying hens in the NF group was less during the replacement period. Morales et al. (18) showed that T-SOD activity increased significantly under starvation and gradually returned to normal levels after refeeding. The present study showed that in stage I, the T-AOC and T-SOD activity were significantly lower in the NF group, which was the result of eliminating excess free radicals generated in the body due to the stress of restricted feeding. In stage III, the T-AOC and T-SOD activity returned to normal levels.

Small intestine tissues are the main site of digestion and absorption in poultry. Under normal conditions, longer VH, shallower CD, and a larger V/C indicate better digestion and absorption of nutrients in the small intestine (19). The gastrointestinal tract of chickens is very responsive to stressors (fasting, temperature changes, etc.), and once stressed, the intestinal epithelial integrity and microbiota are immediately altered (20–22). Numerous studies have found that fasting leads to a significant reduction in duodenal VH (23, 24). Yamauchi et al. (25) also found that during fasting, the reduction in VH in laying hens was very rapid in the first 24 h, followed by a gradual and slow reduction, and a significant increase in VH after resumption of feeding. In the present study, it was shown that in stage I, both the VH and V/C of the small intestine tissues were significantly reduced, but were gradually restored with the resumption of basal feeding. In stage III, the CD of small intestine was significantly decreased, and V/C was increased, indicating that non-fasting molting could improve the intestinal function to some extent. Furthermore, it has been shown that dietary fiber can be fermented to some extent by microorganisms in the gut, and this fermentation can maintain normal digestion and absorption in the gastrointestinal tract and act as a defense against diseases and pathogenic bacteria (26, 27). Therefore, the use of low-energy, low-protein, high-fiber diet for molting may be more beneficial to the intestinal health of laying hens.

ALT and AST are common indicators of animal liver health, and an increase in their activity in plasma usually indicates that the liver has been damaged to varying degrees (28–30). The results of this study showed that in stage I, the ALT and AST activities were significantly higher in the F group than in the NF and C groups. The activities of both enzymes were significantly higher in the NF group than in the C group, and then gradually recovered in stage III when there was no significant difference between the three groups. Studies have shown that fasting or restricted feeding leads to elevated plasma transaminase activity (31, 32); therefore, during the molting period, ALT and AST activities may be associated with fasting or restricted feeding, causing stress in laying hens. At the same time, some studies have found that molting may indirectly affect the activity of plasma transaminases by affecting the target response of steroid hormones (33). Combined with the previous studies of our research group, it can be seen that during the molting period, sex steroid hormones such as progesterone (Prog) and estrogen (E), are significantly reduced, cortical steroids are increased, and plasma ALT and AST activities are indirectly affected, therefore they are significantly increased. VLDL and vitellogenin (Vg) are primarily synthesized by liver cells, providing fatty acids and cholesterol to eggs (34). Studies have shown that there may be a correlation between the egg production rate and plasma VLDL levels. When the egg production rate is low, the plasma VLDL content is correspondingly low; when the egg production rate is high, the plasma VLDL content is also high (35). Walzem et al. (36) also reached the same conclusion in a molting experiment of laying hens. Feed restriction during the molting period resulted in the cessation of egg-laying by laying hens; therefore, the plasma VLDL content decreased accordingly. Our experiment showed that in stage I, as the hens stopped laying eggs, the plasma VLDL content in the NF and F groups also decreased significantly, and then gradually returned to normal levels with an increase in the laying rate.

Weight reduction is one of the necessary processes in all molting procedures, and ~1/4 of the weight reduction comes from the weight of the liver, ovaries, and oviducts (37–40). Grals et al. (41) experimented on 9-month- old rats and found that when feeding was controlled to 60%, the average body weight slowly decreased by 30% and the weight of the liver was also reduced, but after the recovery of free feeding, the increase in feed intake also stimulated liver growth. Therefore, the average body weight and liver weight returned to normal in ~1 week. Landers et al. (42) showed that during the molting period, liver weight was significantly lower in both groups of molting hens than in the control group, but there was no significant difference in liver weight between the fasting and alfalfa-fed groups. The fat content in the liver gradually accumulates with age, and excessive fat can be a burden to the liver. Studies have shown that the fat content in the liver is significantly reduced after molting, which can reduce the burden on the liver and thus improve production performance (39). The results of this experiment are consistent with the above-mentioned studies, in which the liver weight of laying hens in the NF and F groups significantly decreased in stage I, and significantly increased with the resumption of the basal diet; in stage III, there was no significant difference between NF group, F group and C group. Furthermore, it has been shown that a shortened light duration and ovarian degradation also decrease liver weight (11).

According to the pathological section of liver tissue, in stage I, the livers of the two groups of concentrated molting hens in this experiment were damaged to a certain extent; among them, the hepatocytes in the F group were more severely swollen, with unclear intercellular boundaries and slight cytoplasmic infection, whereas the hepatocytes in the NF group had intercellular boundaries, but cytoplasmic infection had started to appear. This result was also supported by the above-mentioned serum biochemical indices of AST and ALT activities, which indicated that the increase in both enzyme activities in the F group was significantly higher than that in the NF group during stage I. The liver recovered gradually with the resumption of the basal diet, and in stage III, the livers of both the NF and F groups had recovered completely, indicating that the molting technique does not cause irreversible damage to the liver of laying hens. Meng et al. (43) also concluded the same conclusion, which indicated that forced molting would not cause irreversible damage to the liver of laying hens. However, according to the serum biochemical indexes and liver sections during the molting period, the non-fasting method was used to molt the laying hens. The degree of damage to the liver is smaller and more conducive to the health of laying hens.

IgG is the most abundant immunoglobulin in the serum of laying hens, and mainly plays antibacterial, antiviral, and neutralizing roles against bacterial toxins, followed by IgA, which plays an important role in resistance to microbial invasion, and IgM, the least abundant initial immune antibody, which protects against pathogens (44). Immunoglobulin levels in the serum of laying hens decreased during the molting period and gradually increased after the resumption of feeding (45). Alondan and Mashaly (6) showed that the immune performance of laying hens during the molting period was suppressed, presumably because prolonged starvation caused stress in laying hens and produced large amounts of CORT, leading to the degeneration of lymphoid tissue, thereby suppressing cellular and humoral immunity (46). Sandhu et al. (45) showed that molting increased immunoglobulin content and improved the immune performance of late-laying hens. The results of this experiment showed that, in stage I, the IgG content of the NF and F groups decreased significantly. With the recovery of the basal diet, IgG content gradually recovered. In stage III, compared with the C group, the IgG content of the NF and F groups increased to a certain extent, but did not reach a significant level. The IgA and IgM contents first decreased and then increased during the three periods.

## 5. Conclusion

In summary, non-fasting molting is less stressful to hens, causes less liver damage, can improve the production performance of hens in the second egg laying cycle, and can improve the intestinal digestion and absorption capacity of laying hens. Therefore, non-fasting molting can improve production efficiency by ensuring animal welfare, which is of great significance in laying hen production facilities.

## Data availability statement

The original contributions presented in the study are included in the article/supplementary material, further inquiries can be directed to the corresponding author.

## Ethics statement

The animal study was reviewed and approved by Animal Use and Ethical Committee of Hebei Agricultural University.

## Author contributions

ML, LS, CH, and FX: designed and completed the experiment. ML, LS, HC, and BZ: statistics and contributions. YC, DW, EH, JZ, HC, and YY: provided experimental guidance. All authors contributed to the article and approved the submitted version.
